# Supplementary material for: Impact of a surgical ward breakfast buffet on nutritional intake in postoperative patients: A prospective cohort pilot study
Source: PLoS One. 2022 Apr 28;17(4):e0267087. doi: 10.1371/journal.pone.0267087 (PMC9049340; doi:10.1371/journal.pone.0267087)
Supplement: S1 Table — Complete case analysis. NA, not applicable; N, number of patients; SD, standard deviation; IQR, interquartile range. Specialism type: HPB, hepatic/pancreatic/biliary; OMS, oral maxillofacial surgery; BMI, body mass index; ASA, American Society of Anesthesiologists Physical Status classification; SNAQ, Short Nutritional Assessment Questionnaire; JH fall risk, Johns Hopkins fall risk assessment; AMEXO, Amsterdam UMC Extension of the Johns Hopkins Highest Level of Mobility scale; DOS, Delirium Observation Scale; NRS0, Numeric Rating Scale at baseline (0).a At risk of undernutrition when score ≥ 3. b Percentage of buffet use per patient during the entire study period. c Number of symptoms during the seven-day study period (e.g. lack of appetite, nausea, full stomach, food tasting different, difficulty chewing or swallowing). d Median pain score during study period. e Percentage of days with a liquid diet during study period. (DOCX) [file pone.0267087.s001.docx]

**S1 Table. Baseline characteristics of the cohort.** Complete case analysis.

| Variables | NA, n (%) | (N = 77) | |
| --- | --- | --- | --- |
| Gender, n (%) |  |  |  |
| Male |  | 38 | (49.4) |
| Age in years, mean (SD) |  | 58.2 | (13.9) |
| Length of stay, median (IQR) |  | 6 | (4 – 9) |
| Department, n (%) |  |  |  |
| A |  | 60 | (77.9) |
| B |  | 17 | (22.1) |
| Specialism type, n (%) |  |  |  |
| HPB |  | 25 | (32.5) |
| Colorectal |  | 27 | (35.1) |
| Esophageal |  | 5 | (6.5) |
| Neuroendocrine |  | 7 | (9.1) |
| Abdominal wall |  | 5 | (6.5) |
| Reconstructive surgery |  | 2 | (2.6) |
| OMS |  | 6 | (7.8) |
| Admission type, n (%) |  |  |  |
| Elective |  | 66 | (85.7) |
| Unplanned |  | 11 | (14.3) |
| Patient undergoing surgery, n (%) |  |  |  |
| Yes |  | 65 | 84.4) |
| BMI (kg/m^2^), mean (SD) | 9 (11.7) | 25.9 | (15.6) |
| Weight (kg), mean (SD) | 1 (1.3) | 80.8 | (4.8) |
| ASA, n (%)^*^ | 16 (20.1) |  | (17.8) |
| ASA I |  | 5 | (6.5) |
| ASA II |  | 47 | (61.0) |
| ASA III |  | 13 | (16.9) |
| SNAQ, n (%)^a^ | 1 (1.3) |  |  |
| Not at risk |  | 71 | (93.0) |
| At risk ≥ 3 |  | 5 | (6.5) |
| JH Fall risk, n (%) |  |  |  |
| Yes |  | 8 | (10.4) |
| No |  | 69 | (89.6) |
| AMEXO, median (IQR) | 17 (22.1) | 8 | (7 – 10) |
| DOS, median (IQR) |  | 0 | (0 – 0) |
| NRS0, median (IQR) | 6 (7.8) | 0 | (0 – 3) |
| Percentage use of the breakfast buffet, median (IQR)^b^ |  | 50 | (0 – 83.3) |
| Number of gastrointestinal symptoms, median (IQR)^c^ |  | 0.7 | (0 – 1) |
| NRS1-7, mean (IQR)^d^ |  | 3.0 | (2 – 4) |
| Liquid diet, mean (SD)^e^ |  | 18.8 | (33.8) |

Complete case analysis.

NA, not applicable; N, number of patients; SD, standard deviation; IQR, interquartile range. Specialism type: HPB, hepatic/pancreatic/biliary; OMS, oral maxillofacial surgery; BMI, body mass index; ASA, American Society of Anesthesiologists Physical Status classification; SNAQ, Short Nutritional Assessment Questionnaire; JH fall risk, Johns Hopkins fall risk assessment; AMEXO, Amsterdam UMC Extension of the Johns Hopkins Highest Level of Mobility scale; DOS, Delirium Observation Scale; NRS0, Numeric Rating Scale at baseline (0).a At risk of undernutrition when score ≥ 3.

^b^ Percentage of buffet use per patient during the entire study period.

^c^ Number of symptoms during the seven-day study period (e.g. lack of appetite, nausea, full stomach, food tasting different, difficulty chewing or swallowing).

**^d^** Median pain score during study period.

**^e^** Percentage of days with a liquid diet during study period.
