# Supplementary material for: Impact of a surgical ward breakfast buffet on nutritional intake in postoperative patients: A prospective cohort pilot study
Source: PLoS One. 2022 Apr 28;17(4):e0267087. doi: 10.1371/journal.pone.0267087 (PMC9049340; doi:10.1371/journal.pone.0267087)
Supplement: S2 Table — Complete case analysis. Complete case analysis. β, beta coefficient; CI, confidence interval; SE, standard error; p, p-value; ASA, American Society of Anesthesiologists Physical Status classification. Specialism: Abd. Wall, abdominal wall surgery; CR, colorectal surgery; HPB, hepatic/pancreatic/biliary surgery; OMS, oral maxillofacial surgery; NE, neuroendocrine surgery; OES, esophageal surgery; Rec., reconstructive surgery; SNAQ, Short Nutritional Assessment Questionnaire; DOS, Delirium Observation Scale; AMEXO, Amsterdam UMC Extension of the Johns Hopkins Highest Level of Mobility scale; JH fall risk, Johns Hopkins fall risk assessment; NRS, Numeric Rating Scale. a Percentage of buffet use per patient during the study period. b Mean pain score during the study period. c Mean number of gastrointestinal symptoms during the seven-day study period (e.g., lack of appetite, nausea, full stomach, food tasting different, difficulty chewing or swallowing). d Percentage of having a liquid diet over the study period. (DOCX) [file pone.0267087.s002.docx]

**S2 Table. The univariate relationship between potential prognostic factors and protein, energy intake.** Complete case analysis.

|  | Protein | | | | | Energy | | | | |
| --- | --- | --- | --- | --- | --- | --- | --- | --- | --- | --- |
|  | **β** | **SE** | **95% CI** | | ***p*** | **β** | **SE** | **95% CI** | | ***p*** |
| Potentially prognostic variable |  |  | **Lower bound** | **Upper bound** |  |  |  | **Lower bound** | **Upper bound** |  |
| Percentage of use of the breakfast buffet^a^ | 0.05 | 0.02 | 0.00 | 0.09 | <0.01 | 0.98 | 0.43 | 0.13 | 1.84 | 0.02 |
| Age | 0.11 | 0.07 | 0.03 | 0.24 | 0.12 | 0.11 | 0.07 | -0.03 | 0.24 | 0.12 |
| Gender, female | -1.14 | 1.92 | -4.98 | 2.69 | 0.56 | -1.14 | 1.92 | -4.98 | 2.69 | 0.56 |
| Weight | 0.09 | 0.05 | -0.01 | 0.20 | 0.08 | 0.09 | 0.05 | -0.01 | 0.20 | 0.08 |
| Type of admission, unplanned | 2.60 | 2.74 | -2.85 | 8.06 | 0.35 | 49.76 | 51.40 | -52.60 | 152.15 | 0.34 |
| ASA | 3.62 | 1.96 | -0.31 | 7.54 | 0.07 | 40.16 | 34.32 | -28.42 | 108.74 | 0.25 |
| Length of stay | -0.04 | 0.17 | -0.39 | 0.30 | 0.80 | -0.04 | 0.17 | -0.39 | 0.30 | 0.80 |
| Surgery specialism |  |  |  |  | 0.14 |  |  |  |  | 0.58 |
| Abd. wall | 12.64 | 3.66 | 5.33 | 19.94 | <0.01 | 289.12 | 70.30 | 149.9 | 429.3 | <0.01 |
| CR | 1.73 | 3.99 | -6.22 | 9.67 | 0.67 | 41.90 | 76.54 | 110.7 | 194.5 | 0.59 |
| HPB | 3.45 | 4.01 | -4.54 | 11.45 | 0.39 | 73.60 | 77.01 | 80.0 | 227.2 | 0.34 |
| OMS | -4.97 | 4.96 | -14.86 | 4.92 | 0.31 | -31.03 | 77.01 | -220.9 | 158.8 | 0.75 |
| NE | 6.47 | 4.80 | -3.09 | 16.03 | 0.18 | 53.67 | 95.19 | 129.9 | 237.3 | 0.56 |
| OES | -1.46 | 5.18 | -11.79 | 8.87 | 0.78 | -49.28 | 99.42 | -247.6 | 149.0 | 0.62 |
| Rec. | 9.28 | 6.85 | -4.38 | 22.94 | 0.18 | 205.55 | 131.52 | -56.8 | 467.9 | 0.12 |
| SNAQ | 1.55 | 0.79 | -0.04 | 3.13 | 0.06 | 18.16 | 15.15 | -12.03 | 48.35 | 0.23 |
| DOS | -9.55 | 5.96 | -21.42 | 2.32 | 0.11 | -241.93 | 110.30 | -461.67 | -22.19 | 0.03 |
| AMEXO | 0.80 | 0.40 | -0.01 | 1.61 | 0.05 | 11.52 | 7.76 | -4.02 | 27.06 | 0.14 |
| JH fall risk, no risk | 2.59 | 3.15 | -3.68 | 8.85 | 0.41 | 75.75 | 57.67 | -41.12 | 192.62 | 0.20 |
| Mean NRS pain score^b^ | -0.27 | 0.63 | -1.52 | 0.99 | 0.67 | -4.13 | 11.77 | -27.58 | 19.32 | 0.73 |
| Mean number of gastrointestinal symptoms^c^ | -3.25 | 1.37 | -5.98 | -0.52 | 0.02 | -1.50 | 1.70 | -4.93 | 1.94 | 0.38 |
| Percentage of having a liquid diet^d^ | -0.07 | 0.03 | -0.13 | -0.02 | 0.01 | -0.81 | 0.53 | -1.86 | -0.25 | 0.13 |

Complete case analysis.

β, beta coefficient; CI, confidence interval; SE, standard error*; p,* *p*-value; ASA, American Society of Anesthesiologists Physical Status classification. Specialism: Abd. Wall, abdominal wall surgery; CR, colorectal surgery; HPB, hepatic/pancreatic/biliary surgery; OMS, oral maxillofacial surgery; NE, neuroendocrine surgery; OES, esophageal surgery; Rec., reconstructive surgery; SNAQ, Short Nutritional Assessment Questionnaire; DOS, Delirium Observation Scale; AMEXO, Amsterdam UMC Extension of the Johns Hopkins Highest Level of Mobility scale; JH fall risk, Johns Hopkins fall risk assessment; NRS, Numeric Rating Scale.

**^a^** Percentage of buffet use per patient during the study period.

**^b^** Mean pain score during the study period.

**^c^** Mean number of gastrointestinal symptoms during the seven-day study period (e.g., lack of appetite, nausea, full stomach, food tasting different, difficulty chewing or swallowing).

**^d^** Percentage of having a liquid diet over the study period.
