# Supplementary material for: Impact of a surgical ward breakfast buffet on nutritional intake in postoperative patients: A prospective cohort pilot study
Source: PLoS One. 2022 Apr 28;17(4):e0267087. doi: 10.1371/journal.pone.0267087 (PMC9049340; doi:10.1371/journal.pone.0267087)
Supplement: S3 Table — Complete case analysis. Complete case analysis. β, beta coefficient; CI, confidence interval; p, p-value; DOS, Delirium Observation Scale. a Percentage of use buffet use per patient during the study period. (DOCX) [file pone.0267087.s003.docx]

**S3 Table. The multivariable regression analyses of prognostic factors for protein, energy intake.** Complete case analysis.

|  | **Protein** | | | | | **Energy** | | | | |
| --- | --- | --- | --- | --- | --- | --- | --- | --- | --- | --- |
| **Prognostic variables** | **β** | **SE** | **95% CI** | | ***p*** | **β** | **SE** | **95% CI** | | ***p*** |
|  |  |  | **Lower bound** | **Upper bound** |  |  |  | **Lower bound** | **Upper bound** |  |
| Percentage of use of the breakfast buffet^a^ | 0.05 | 0.02 | 0.01 | 11.87 | 0.02 | 1.00 | 0.41 | 0.17 | 1.82 | 0.02 |
| Weight | 0.11 | 0.05 | 0.00 | 0.21 | 0.04 |  |  |  |  |  |
| SNAQ | 1.27 | 0.64 | 0.00 | 2.55 | 0.05 |  |  |  |  |  |
| DOS |  |  |  |  |  | -246.29 | 106.95 | -459.38 | -33.19 | 0.02 |
| Multiple linear regression model protein: R^2^ = 0.15, adjusted R^2^ = 0.11 | | | | | | | | | | |
| Multiple linear regression model energy: R^2^ = 0.13, adjusted R^2^ = 0.11 | | | | | | | | | | |

Complete case analysis.

β, beta coefficient; CI, confidence interval; *p*, *p*-value; DOS, Delirium Observation Scale.

**^a^** Percentage of use buffet use per patient during the study period.
