# Supplementary material for: Impact of a surgical ward breakfast buffet on nutritional intake in postoperative patients: A prospective cohort pilot study
Source: PLoS One. 2022 Apr 28;17(4):e0267087. doi: 10.1371/journal.pone.0267087 (PMC9049340; doi:10.1371/journal.pone.0267087)
Supplement: S1 File — Dutch version. (DOCX) [file pone.0267087.s004.docx]

**S1 File. Patient self-report diary**. Dutch version.

| ***DAG 1:* Eet – en drink dagboek** | | |  |
| --- | --- | --- | --- |
| 1. Welke datum is het vandaag? | ________--________--________ | |  |
| 1. Van welke ontbijtservice heeft u vanochtend gebruik gemaakt? | Bij ontbijtbuffet  Reguliere ontbijtservice | |  |
| 1. Had u tijdens het ontbijt één of meer klachten die het eten en drinken moeilijk maakten? | Minder zin in eten  Misselijk  Snel een vol gevoel  Eten smaakt anders  Moeilijk kauwen of slikken  Geen klachten  Anders, namelijk:  _____________________________________ | |  |
| 1. Had u tijdens het ontbijtmoment een vloeibaar dieet? | Ja  Nee | |  |
| **Geef aan hoeveel en welke producten u heeft gegeten en gedronken tijdens het ontbijt:** | | | |
| **Brood** | | **Yoghurtbar & Topping** | |
| Dikke snee topfit meergranen  Fijn volkoren boterham  Luchtige volkoren cracker  Witte boterham  Flensje  Rozijnenbrood  Beschuit  Eierkoek | | Magere kwark  Griekse yoghurt  Walnoten  Appel  Fruit  Pompoenpitten  Muesli  Honing  Kaneel  Chocolade puur  Dessertsaus aardbei | |

| **Pap** | **Broodbeleg** | | |
| --- | --- | --- | --- |
| Havermoutpap, eiwitrijk  Cornflakes met melk | Gekookt scharrelei  Smeerkaas 48+  Kees kaas extra gerijpt  Petit paté (vegetarisch)  Komijnekaas 20+  Hummus  Jong belegen kaas 48+  Chocoladehagelslag  Eiersalade  Aardbeienjam  Runderrookvlees  Pindakaas  Kipfilet  Honing  Runderpastrami (Halal)  Appelstroop  Gebraden gehakt  Dieethalvarine  Schouderham  Botermelange | | |
| **Smaakmakers** |  |  |  |
| Appelstroop  Chutney  Ketchup  Piccalilly  (Mosterd) mayonaise  Suiker  Peper en zout |  |  |  |
| **Dranken** | | | **Melk, yoghurt & vla** |
| Koffie  Thee  Sinaasappelsap  Appelsap  Mineraal bruisend  Limonade framboos 0.0  Limonade sinaasappel  Huisgemaakt kruiden-/fruitwater | | | Halfvolle melk  Karnemelk  Chocolademelk  Sojadrink  Vanillevla |
| **Fruit** | | **Eiwit- en energierijke drinkvoeding** | |
| Sinaasappel  Appel  Banaan  Appelmoes  Overig:  __________________________  __________________________  __________________________ | | Nutridrink Compact Proteïne  Nutridrink Compact  Nutridrink Yoghurt Style  Nutridrink Juice Style  Nutridrink Smoothie zomerfruit  Diasip  **Vloeibaar dieet**: Nutridrink crème vanille  **Vloeibaar dieet**: Nutridrink crème chocolade | |
