# Supplementary material for: Impact of a surgical ward breakfast buffet on nutritional intake in postoperative patients: A prospective cohort pilot study
Source: PLoS One. 2022 Apr 28;17(4):e0267087. doi: 10.1371/journal.pone.0267087 (PMC9049340; doi:10.1371/journal.pone.0267087)
Supplement: S2 File — English version. (DOCX) [file pone.0267087.s005.docx]

**S2 File. Patient self-report diary**. English version.

| ***Day 1:* Eat - and drink diary** | | |  |
| --- | --- | --- | --- |
| 1. What date is today? | ________--________--________ | |  |
| 1. Which breakfast service did you use this morning? | Breakfast buffet  Regular breakfast service | |  |
| 1. Did you experience symptoms that could limit intake during breakfast? | Lack of appetite  Nausea  Full stomach  Food tastes different  Difficulty chewing or swallowing  No symptoms  Other symptoms, namely:  _____________________________________ | |  |
| 1. Did you have a liquid diet during breakfast? | Yes  No | |  |
| **Indicate how many and which products you ate and drank during breakfast:** | | | |
| **Bread** | | **Yogurt bar & Toppings** | |
| Slice of multigrain bread  Wholemeal sandwhich  Light whole wheat cracker  Slice of white bread  Warm crepe  Slice of raisin bread  Rusk  ‘*Eierkoek’* | | Low-fat quark  Greek yogurt  Walnuts  Apple  Fruit  Pumpkin seeds  Muesli  Honey  Cinnamon  Dark chocolate  Dessert sauce strawberry | |

| **Porridge** | **Bread toppings** | | |
| --- | --- | --- | --- |
| Protein oatmeal porridge  Cornflakes with milk | Boiled egg  Cheese spread 48+  Cheese slices  Pâté (vegetarian)  Cumin cheese 20+  Hummus  Young cheese 48+  Chocolate *‘hagelslag´*  Egg salad  Strawberry Jelly  Smoked beef  Peanut butter  Chicken fillet  Honey  Pastrami (Halal)  Apple syrup  Roasted minced meat  Low-fat margarine  Pork  Butter melange | | |
| **Seasonings** |  |  |  |
| Apple syrup  Chutney  Ketchup  Piccalilly  (Mustard) mayonnaise  Sugar  Pepper and salt |  |  |  |
| **Drinks** | | | **Milk, yogurt & custard** |
| Coffee  Tea  Orange juice  Apple juice  Mineral sparkling water  Lemonade - raspberry 0.0  Lemonade - orange  Homemade herbal-/fruit water | | | Semi-skimmed milk  Buttermilk  Chocolate milk  Soja drink  Vanilla custard |
| **Fruit** | | **Protein- and energy-rich drinks** | |
| Orange  Apple  Banana  Apple sauce  Other:  __________________________  __________________________  __________________________ | | Nutridrink Compact Proteïne  Nutridrink Compact  Nutridrink Yogurt Style  Nutridrink Juice Style  Nutridrink Smoothie ‘*zomerfruit’*  *Diasip*  **Liquid diet**: Nutridrink creme vanilla  **Liquid diet**: Nutridrink creme chocolate | |
